# Coincidence of Sarcoidosis and a COVID-19 vaccine-associated hypermetabolic lymphadenopathy in a patient with a history of invasive breast cancer: A case report

**DOI:** 10.1016/j.ijscr.2022.107098

**Published:** 2022-04-19

**Authors:** Asuka Kawabata, Toru Nakamura, Hanae Suzuki, Masayuki Yoshida, Yoshiro Otsuki, Natsuko Mori

**Affiliations:** a Department of Breast Surgery, Seirei Hamamatsu General Hospital, 2-12-12 Sumiyoshi, Nakaku, Hamamatsu-city, Shizuoka 430-8558, Japan; b Department of General Thoracic Surgery, Seirei Hamamatsu General Hospital, 2-12-12 Sumiyoshi, Nakaku, Hamamatsu-city, Shizuoka 430-8558, Japan; c Department of Pathology, Seirei Hamamatsu General Hospital, 2-12-12 Sumiyoshi, Nakaku, Hamamatsu-city, Shizuoka 430-8558, Japan

**Keywords:** BHL, bilateral hilar lymphadenopathy, VAHL, vaccine-associated hyper metabolic lymphadenopathy, EBUS-TBNA, endobronchial ultrasound-guided trans-bronchial needle aspiration, Breast cancer, Sarcoidosis, Vaccine-associated hypermetabolic lymphadenopathy

## Abstract

**Introduction and importance:**

Vaccine-associated hypermetabolic lymphadenopathy (VAHL) after a COVID 19 vaccination is a common adverse event and also a diagnostic challenge especially in patients with a history of a malignancy.

**Case presentations:**

A 47-year-old woman presented with enlarged lymph nodes in the right hilar, subcarinal, and right supraclavicular regions detected by computed tomography as a postoperative follow-up study of thyroid cancer. Fluorine-18 fluoro-2-deoxy-d-glucose positron emission tomography (FDG-PET) performed 3 weeks later revealed an FDG uptake in those swollen lymph nodes and in the novel lymphadenopathy in the left axilla and left subclavicular regions. Both biopsy specimens from the right supraclavicular and hilar lymph nodes revealed only multiple small granulomas with multinucleated giant cells without malignancy, consistent with sarcoidosis. The left axilla and subclavicular lymphadenopathy detected by the FDG-PET subsequently spontaneously regressed.

**Clinical discussion:**

The coincidental occurrence of VAHL and lymphadenopathy in sarcoidosis patients could cause diagnostic confusion especially in those with breast cancer.

**Conclusion:**

Sufficient attention should be paid both to the injection site and the time interval between the vaccination and imaging test in the era of nationwide mass vaccinations against COVID 19.

## Highlights

• Vaccine-associated hypermetabolic lymphadenopathy (VAHL) after a COVID 19 vaccination is a common adverse event.
• Sarcoidosis is a multisystem granulomatous disorder of an unknown etiology and often involves the lymph nodes.
• The coincidence of VAHL and sarcoidosis poses a diagnostic challenge in patients with a history of breast cancer. Attention should be paid to both the injection site and time interval between the vaccination and oncological imaging test in the era of widespread COVID-19 vaccinations.

## 1 Introduction

Sarcoidosis is a multisystem granulomatous disorder of unknown etiology. It often involves the lymph nodes, and a bilateral hilar lymphadenopathy (BHL) is a characteristic imaging finding [1]. Tuberculous lymphadenitis, malignant lymphomas, and lymph node metastases from any primary site could also manifest as a BHL [2]. Mediastinal lymph node metastasis recurrences are less frequent but can occur in 2% of patients with breast cancer [3]. Swollen lymph nodes after COVID 19 vaccinations are a common adverse event and could be a diagnostic challenge especially in patients with a history of a malignancy [4].

We report a case of a COVID19 vaccine-associated hyper metabolic lymphadenopathy (VAHL) concomitant with sarcoidosis in a patient with breast cancer.

This work has been reported in line with the SCARE criteria [5].

## 2 Case presentation

**Fig. 1:**
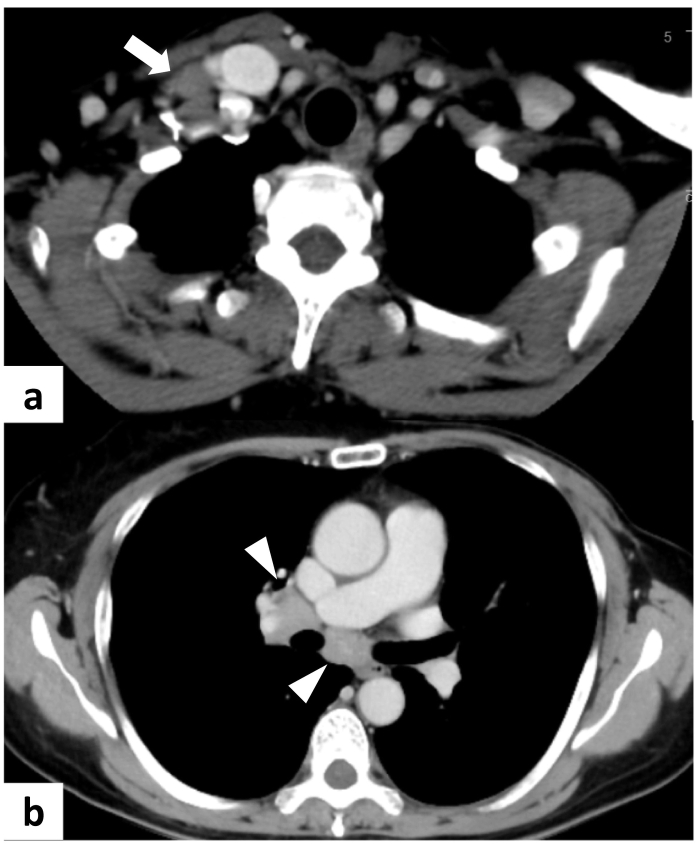
a: A contrast-enhanced chest CT scan showing enlarged lymph nodes located in the right supraclavicular region (arrow). b: The enlarged lymph nodes were also visualized in the right hilar and subcarinal regions (arrow heads).

A 47-year-old woman presented with enlarged lymph nodes in the right hilar, subcarinal, and right supraclavicular regions detected by computed tomography (CT) as a follow-up study of thyroid cancer (Fig. 1). She had undergone a left mastectomy for Luminal B breast cancer 7 years prior and a thyroidectomy for a papillary thyroid microcarcinoma 4 years prior. Post-examination questioning revealed that she had the first dose of the Pfizer-BioNTech COVID-19 vaccine (BNT162b2) in her left upper extremity 2 days prior to the CT scan.

**Fig. 2:**
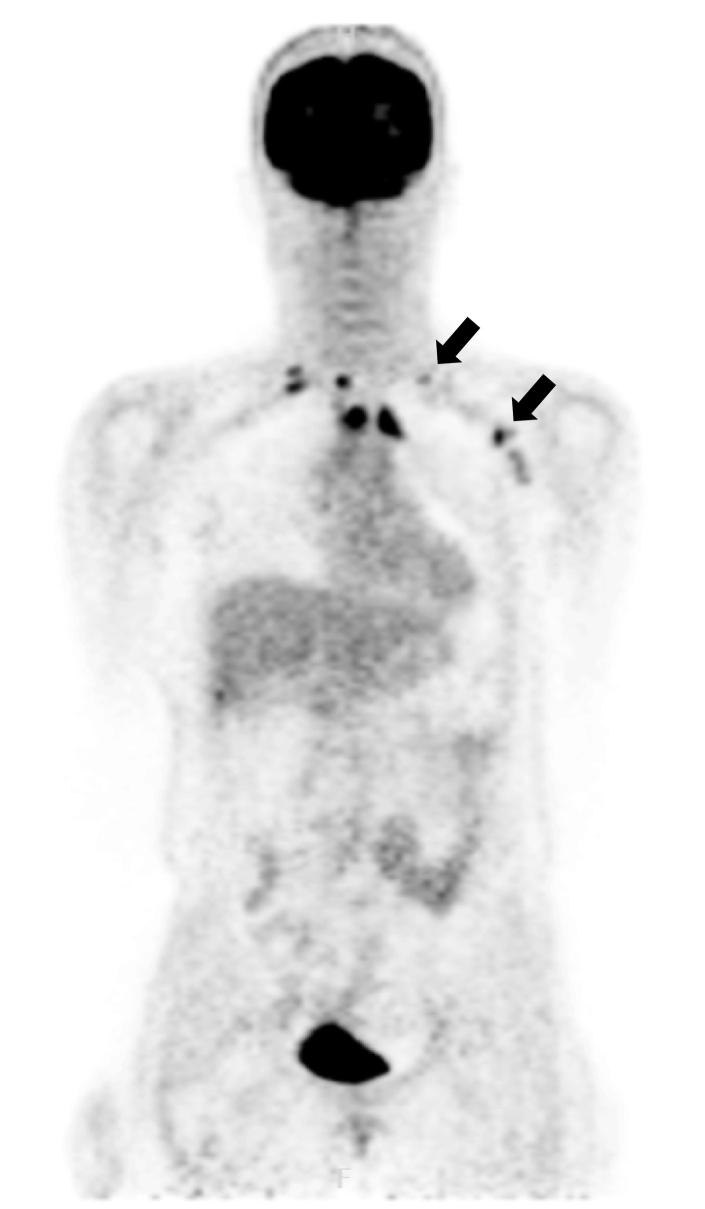
FDG-PET showing the novel lymphadenopathy with an FDG uptake in the left axilla and left supraclavicular region (arrows).

Fluorine-18 fluoro-2-deoxy-d-glucose positron emission tomography (FDG-PET) performed 3 weeks later revealed an FDG uptake in those swollen lymph nodes and in the novel lymphadenopathy in the left axilla and left subclavicular regions (Fig. 2).

Since the patient had received a second dose of the COVID19 vaccine 3 days prior to the PET imaging, the novel lymphadenopathy might have been caused by the vaccination. However, it also should be distinguished from a malignant lymphoma because of the rapid progression.

**Fig. 3:**
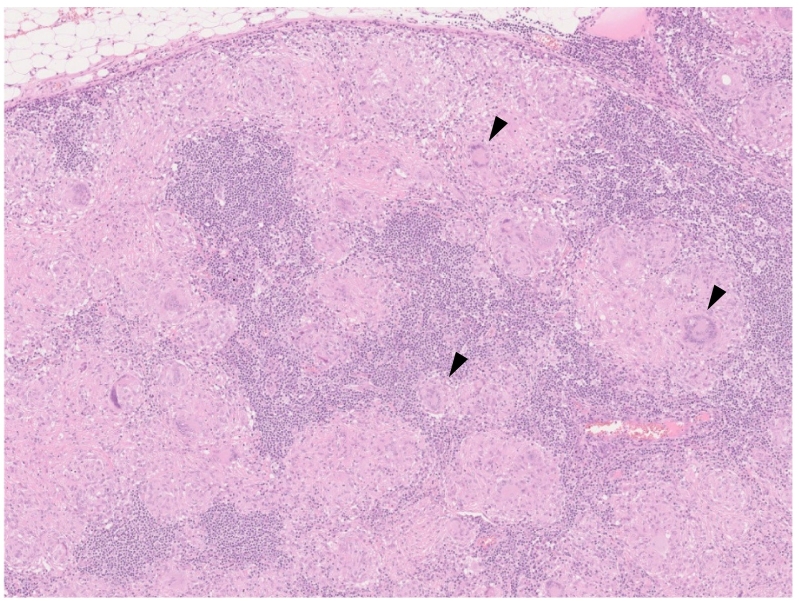
A histopathological examination of the right supraclavicular lymph node revealed multiple granulomatous lesions with multinucleated giant cells (arrow heads).

She underwent an open biopsy of the right supraclavicular lymph node under general anesthesia. The histology revealed multiple granulomas with multinucleated giant cells without any malignancy (Fig. 3). A further histological assessment from the hilar lymph node deemed essential to identify sarcoidosis from a sarcoid reaction associated with a malignant lymphoma.

**Fig. 4:**
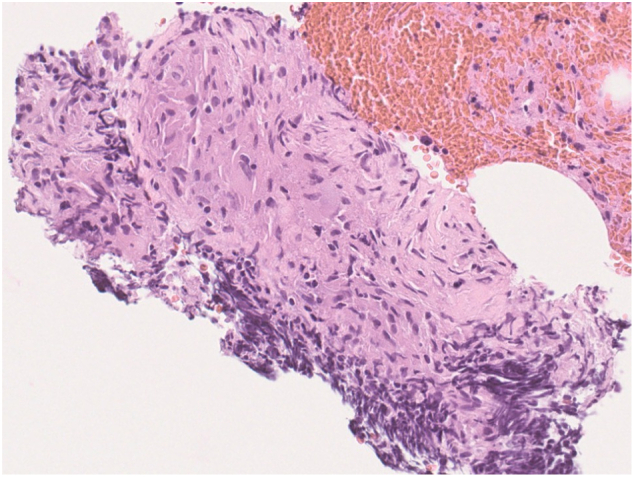
A histopathological examination of the right hilar lymph node revealed granulomatous lesions characteristic of sarcoidosis without malignancy.

An endobronchial ultrasound-guided trans-bronchial needle aspiration (EBUS-TBNA) was performed on the right hilar lymph node through a 21-gauge needle and the histology showed an epithelioid granuloma along with lymphoid tissue (Fig. 4). Whereas serum angiotensin converting enzyme and lysozyme levels were normal, those results suggested that more than one organ system was involved from the granulomas without malignancy, consistent with the diagnosis of sarcoidosis [6].

An additional echocardiography and ophthalmologic examination revealed no ocular or cardiac involvement, suggesting no therapeutic requirements. The left axillary and subclavicular lymphadenopathy regressed spontaneously and continued to be undetectable by ultrasonography at 7 months after the PET imaging.

## 3 Discussion

Sarcoidosis is a multisystem granulomatous disorder of unknown etiology that could involve any organ such as the lymph nodes, lungs, eyes, or skin [1]. FDG-PET imaging is usually performed to evaluate its activity and treatment effects. The term “sarcoid like reaction” represents the radiological and histological characteristics mimicking sarcoidosis in patients with an underlying malignancy [7]. They could develop in a variety of malignancies such as testicular tumors, lymphomas, or lung, stomach, uterine, or breast cancers, and could be radiologically indistinguishable from metastases because of the FDG uptake [8], [9], [10]. A malignant lymphoma was highly suspected during the initial work up in the present case because of its rapid progression during the repeat radiological tests. The histopathological findings of both the supraclavicular and additional hilar lymph node biopsy confirmed the diagnosis of sarcoidosis. Mediastinoscopy is a traditional diagnostic modality for hilar and mediastinal lymph nodes but is also associated with surgical morbidities such as a hemorrhage, pneumothorax, or laryngeal nerve injury [11], [12]. In recent years, EBUS has become popular because of its less invasiveness and accuracy, and it was diagnostic in the present case [13].

VAHL after a COVID19 vaccination (BNT162b2/1273) is a common adverse event and the most frequently involved site is the axilla, followed by the supraclavicular, neck, and pectoral regions [14], [15], [16], [17], [18]. It poses a diagnostic challenge in the FDG-PET with which both the benign and malignant nature could reveal a hyper metabolic lymphadenopathy. It is more prevalent in women and a vaccination on the affected side of patients with breast cancer could yield a VAHL mimicking a lymph node metastasis [19]. Furthermore, a VAHL is more frequent after a booster vaccination than after the first dose with the highest incidence and grade during the first 6 days [20]. Consideration should have been given to the injection site and the time interval between the booster dose and PET imaging to avoid the diagnostic confusion in the present case.

## 4 Conclusion

A coincidental occurrence of a VAHL and lymphadenopathy in patients with sarcoidosis could cause diagnostic confusion especially in those with breast cancer. An EBUS-TBNA is a feasible option for a histological confirmation of a hilar lymph node. Sufficient attention should be paid to both the injection site and time interval between the vaccination and oncological imaging test in the era of nationwide mass vaccinations against COVID 19.

## Funding

Not applicable.

## Consent

Written informed consent was obtained from the patient for publication of this case report and accompanying images. A copy of the written consent is available for review by the Editor-in-Chief of this journal on request.

## Ethical approval

Not applicable.

## Consent

Written informed consent was obtained from the patient for publication of this case report and accompanying images. A copy of the written consent is available for review by the Editor-in-Chief of this journal on request.

## Author contribution

AK wrote this paper. YO reviewed the pathological findings. All authors read and approved the final manuscript.

## Registration of research studies

Not applicable.

## Guarantor

Masayuki Yoshida.

## Availability of data and material

Not applicable.

## Provenance and peer review

Not commissioned, externally peer-reviewed.

## Declaration of competing interest

The authors declare that they have no competing interests.
